# An Assessment of Demand for a Combined PharmD–MBA Program at the University of Saskatchewan

**DOI:** 10.3390/pharmacy4020020

**Published:** 2016-05-13

**Authors:** Kerry Mansell, Antoine Bruneau-Bouchard, Vincent Bruni-Bossio

**Affiliations:** 1 College of Pharmacy and Nutrition, University of Saskatchewan, 104 Clinic Place, Saskatoon, SK S7N 5E5, Canada; 2 Edwards School of Business, University of Saskatchewan, 25 Campus Drive, Saskatoon, SK S7N 5A7, Canada; bruneau.bouchard@usask.ca (A.B.-B.); Bruni-bossio@edwards.usask.ca (V.B.-B.)

**Keywords:** pharmacy education, PharmD, MBA, combined degrees

## Abstract

(1) Background: Combined MBA programs are becoming increasingly popular, and it is anticipated that there will be 60 combined pharmacy–MBA programs across North America in 2015. We aimed to see if there would be support for a combined PharmD–MBA program at the University of Saskatchewan. (2) Methods: A questionnaire was distributed to 1st, 2nd, and 3rd year pharmacy students at the University of Saskatchewan. A separate questionnaire was developed and all practicing pharmacists in Saskatchewan were emailed a link to SurveyMonkey^®^ (Palo Alto, CA, USA) to fill it out online. In-person and phone interviews were conducted with pharmacy stakeholders in Saskatchewan and across the country. (3) Results: Of the 265 students, 193 (72.8%) were present on the days the questionnaires were distributed, and they all completed the questionnaires. When asked if they would have pursued a combined degree if the U of S had offered it when they entered the pharmacy program, 16.6% (32/193) and 37.3% (72/193) either strongly agreed or agreed and 29.0% (56/193) were unsure. When pharmacists were asked if an MBA would be valuable or applicable in their current job, 42.2% (128/303) agreed and 13.9% (42/303) strongly agreed. When asked if they felt students graduating with a combined degree would be at an advantage for certain job opportunities upon graduation, 33.6% (100/298) strongly agreed and 55.4% (165/298) agreed. A total of 8 interviews were conducted with key stakeholders from across Canada. Of these 8 stakeholders, only 2 were aware that other combined programs were offered. All of the stakeholders were in favour of the idea of a combined degree. Some felt it was important for the program to have a clear value proposition and healthcare related content would be desirable. (4) Conclusions: Overall, pharmacist, pharmacy student, and stakeholder input indicate that a combined program could be supported at the University of Saskatchewan.

## 1. Introduction

Pharmacy programs offered by Canadian schools are focused on producing high quality pharmacists with strong clinical skills. These clinical skills help manage decision-making processes in order to support therapeutic decisions in the best interests of the patient. However, not all pharmacists pursue careers in direct patient care. Many go on to have administrative positions in large teaching hospitals, work in the pharmaceutical industry, or own their own businesses. Intertwined throughout pharmacy programs are management or business classes, with the intent of providing some exposure to the business of pharmacy prior to entering the profession. However, business and management exposure is typically limited in pharmacy schools [1].

A Master of Business Administration (MBA) is designed to help people develop the skills necessary to be successful in business. Combining an MBA with another degree is attractive for a number of reasons. The business skills learned in an MBA program complement the skills learned in one’s chosen profession so as to provide individuals with the greatest chance of having successful careers. Having both a pharmacy and MBA degree is considered desirable for those looking to work in academia, the pharmaceutical industry or large institutional settings [2]. Whereas an MBA has traditionally been earned after one’s initial degree, there has been a recent trend of offering combined degree programs at the same time. The objectives of combined programs are to provide students with the necessary skills for management positions, and to allow them to earn a degree in less time and for less money than if they were pursued separately [3].

Throughout the past couple of decades, combined MBA degrees have increased in popularity. Between 1993 and 2001 the number of MD/MBA programs in the USA grew from 6 to 33 [4], and as of 2011 this number grew to 65 [5]. PharmD/MBA programs have been offered in the USA for the past twenty years, and it is anticipated that just less than half (58/134) of all US pharmacy schools will offer a combined PharmD/MBA program in 2015-16 [6,7]. Within the last 3 years, two Canadian universities (University of Alberta and University of Toronto) have introduced combined pharmacy–MBA programs. The increasing number of schools offering combined programs aligns with the policy position of the American Society of Health-System Pharmacists (ASHP), who advocate for pharmacy schools to develop more opportunities for combined degrees, with the purpose of fostering leadership and management competencies help fill leadership positions in pharmacy [8].

Although the number of combined MBA programs is increasing and seemingly aligns with the vision of creating leaders in respective professions, to our knowledge, there has never been an assessment of the demand for combined pharmacy–MBA degrees. The merits and demands for MBA degrees across all fields have been debated in today’s business environment [9,10,11]. One small US study found that students in a combined PharmD–MBA program reported high satisfaction with the program, increased career opportunities, and significantly higher compensation upon entering the workforce than their colleagues not in the combined program [2].

Similar to other pharmacy schools across Canada, the College of Pharmacy and Nutrition, University of Saskatchewan is currently undergoing curriculum change as it moves towards offering a PharmD as a first-degree program. As other Canadian pharmacy schools currently offer a combined pharmacy–MBA degree, this study aimed to determine if the pharmacy community felt it was worthwhile for the University of Saskatchewan to offer a combined PharmD–MBA program.

## 2. Methods

A brief questionnaire was developed to determine pharmacy students’ awareness of MBA programs, and if they would have pursued a combined pharmacy–MBA degree if the opportunity had been offered to them. A hard-copy questionnaire was distributed to the 1st, 2nd, and 3rd year pharmacy students at the beginning of one of their lectures (4th year students were away on rotations). The questionnaire consisted of 12 questions which were either yes or no questions or utilized a 5-point Likert-scale where students were instructed to respond if they strongly agree, agree, disagree, strongly disagree, or were unsure. Students also had the ability to provide comments at the end. All questionnaires were completed between 24 March and 7 April 2015, and were completely anonymous with no means of identifying respondents.

A brief questionnaire was developed to determine the opinions of practicing pharmacists in Saskatchewan regarding their assessment of business preparedness and the value of an MBA degree. The questionnaire consisted of 10 questions, plus the ability to provide comments at the conclusion of the questionnaire. These questions utilized a 4-point Likert scale, where pharmacists were asked to respond if they strongly agreed, agreed, disagreed, or strongly disagreed. This was an online questionnaire available for completion through SurveyMonkey^®^. A notice for the study was emailed to all practicing pharmacists for whom the Saskatchewan College of Pharmacists has an email address. The initial email was sent 8 April 2015, and a follow-up email was sent 17 April 2015. The study was closed to responses 6 May 2015.

Phone and in-person interviews were conducted with a few key pre-selected pharmacy stakeholders to determine their familiarity with and opinions on a combined pharmacy–MBA program. These interviews were performed by the same interviewer between 19 March and 6 April 2015. Interviews were semi-structured and consisted of 8 questions. These interviews were assessed by two separate reviewers.

Basic descriptive statistics were performed for the questionnaires. An ethics exemption was granted by the University of Saskatchewan’s Behavioral Research Ethics Board.

## 3. Results

### 3.1. Student Survey

There are 265 pharmacy students in first, second, and third year at the College of Pharmacy and Nutrition, University of Saskatchewan (U of S). Not all students were present on the day the questionnaires were distributed, and overall 193 (72.8%) completed the questionnaires.

The majority of students (190/193; 98.4%) reported that they did not have a previous business degree, and only 4.7% (9/193) have owned or managed a business in the past. Upon graduation, 12.4% (24/193) and 37.8% (73/193), respectively, strongly agreed or agreed that they were interested in owning a pharmacy upon graduation, and 32.1% (62/193) indicated they were unsure. The remaining 17.6% (34/193) either strongly disagreed or disagreed they were interested in owning a pharmacy. Similarly, 11.4% (22/193) and 42.0% (81/193) indicated they strongly agreed or agreed that they were interested in pursuing a career in management, whereas 30.6% (59/193) indicated they were unsure. The remaining 16.1% (31/193) either strongly disagreed or disagreed they were interested in pursuing a career in management.

When asked if students felt their education prepared them for management or ownership positions, 42.0% (81/193) disagreed, 8.8% (17/193) strongly disagreed, 31.1% (60/193) were unsure, and 17.6% (34/193) and 0.5% (1/103) respectively either agreed or strongly agreed. 50.8% (98/193) felt an MBA would help them in their future career, whereas 11.4% (22/193) disagreed and 37.3% (72/193) were unsure.

Only 6.2% (12/193) of students were familiar with other Canadian pharmacy schools that offered combined pharmacy–MBA programs, and only 2.6% (5/193) of students were familiar with pharmacy schools in the United States of America that offered combined programs. When asked if they would have pursued a combined degree if the U of S had offered it when they entered the pharmacy program, 16.6% (32/193) and 37.3% (72/193) either strongly agreed or agreed. 29.0% (56/193) were unsure, and 10.4% (20/193) and 5.7% (11/193) disagreed and strongly disagreed respectively. Upon graduation, 3.1% (6/193) and 11.9% (23/193) strongly agree or agree that they are interested in pursuing an MBA degree, whereas 31.6% (61/193) are still unsure.

### 3.2. Online Survey

The online survey was emailed to 1529 practicing pharmacists in Saskatchewan; 304 completed the questionnaire for a 19.9% response rate. The majority of respondents identified themselves as staff community pharmacists (110/304; 36.2%) and having graduated more than 10 years ago (183/303; 60.4%). A complete list of pharmacist demographics is described in Table 1.

Most respondents strongly disagreed (24.2%; 73/302) or disagreed (54.3%; 164/302) that they were prepared business-wise for their career as a pharmacist upon graduation. When asked if an MBA would be valuable or applicable to their current job, 42.2% (128/303) agreed, 13.9% (42/303) strongly agreed, and 35.6% (108/303) and 8.3% (25/303) either disagreed or strongly disagreed, respectively.

**Figure 1:**
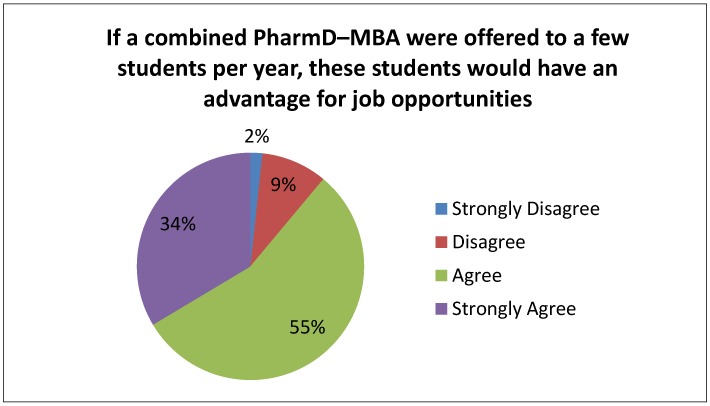
Pharmacist opinions (*n* = 298) on if students graduating with a combined PharmD–MBA would have an advantage for job opportunities.

When asked if they would have been interested in pursuing a combined pharmacy–MBA degree if it had been offered when they were students, 17.6% (53/301) strongly agreed and 39.5% (119/301) agreed (Table 2). When asked if they felt students graduating with a combined degree would be at an advantage for certain job opportunities upon graduation, 33.6% (100/298) strongly agreed and 55.4% (165/298) agreed (Figure 1). Most pharmacists indicated that they strongly disagreed (30.4%; 92/303) or disagreed (52.5%; 159/303) that they were aware of other combined pharmacy–MBA programs in Canada.

### 3.3. Interviews

A total of 8 interviews were conducted with representatives from pharmacy advocacy and regulatory bodies, large institutional (hospital) settings, and large retail chains. Participants were intentionally chosen from varied settings to obtain a broad spectrum of responses; to ensure individual identities are not revealed, all results are presented in aggregate. Thematic analysis and a full qualitative assessment were not performed, as these interviews were simply meant to get a feeling of acceptance or reluctance with respect to the introduction of a combined pharmacy–MBA program.

To begin, these 8 stakeholders were asked if they were aware of any currently offered combined programs in either Canada or the USA. Of them, 2 were aware that other combined programs were offered. When asked, in general, if they felt a combined program was a good idea, all participants indicated yes. Some were emphatically in favour, whereas others felt there should be qualifiers, such as the number of students enrolled, and that it should not detract from patient care.

All of the stakeholders were in favour of the idea of a combined degree. However, 2 respondents wanted to make sure it had a clear value proposition for students and the College of Pharmacy and Nutrition. Further, one respondent felt the number of students allowed to do a combined degree should be limited, and one person wanted to see a program that was affordable. It was also mentioned by one respondent that it should be differentiated from other Canadian schools offering a combined program, and another respondent mentioned that the combined program should bring value to the students and not be just a token add-on program. Overall, the idea of a combined degree was supported, with an overarching sense that it should bring value to the students enrolled.

When asked which type of students they felt would benefit from a combined degree, the majority indicated them to be those looking at ownership or any senior management position. It was mentioned by 2 respondents that higher level positions within large companies and large healthcare institutions now often require an MBA, and that senior level positions would no longer be attainable without one. Respondents also expressed that those entering academia, advocacy organizations, the pharmaceutical industry or doing policy analysis would benefit from a combined degree as well. One respondent felt that any pharmacist would benefit, as there are skills learned in the MBA program that will help make people successful regardless of their position.

Several respondents expressed that pharmacy is only one small piece of the healthcare system, and if pharmacists are to play a larger role, it is vital to understand the business of healthcare and how pharmacists fit in. Numerous respondents mentioned that ‘pharmacy’ is often on the outside looking in, and that pharmacy leaders with business acumen are needed to understand the broader context of health care (particularly within large hospital institutions) and that would be the value of an MBA to pharmacy. When asked if they were able to have any input into the design of the MBA program, the majority of respondents expressed they would like to see some business-course content which is health care related.

Finally, when asked from a high-level view if a combined program is something the College of Pharmacy and Nutrition should consider as they undergo a transition to the PharmD program, all of the respondents indicated yes.

## 4. Discussion

Combined MBA programs are increasing in popularity and are already offered at two pharmacy schools in Canada. With the College of Pharmacy and Nutrition undergoing curriculum redesign towards a PharmD as a first-degree, the timing is ideal to evaluate whether a combined PharmD–MBA program could be supported at the University of Saskatchewan.

The results of the student and pharmacist questionnaires indicate that there is a level of demand for a combined PharmD/MBA program. When asked if they would have pursued a combined pharmacy–MBA degree had it been offered to them, 54% of pharmacy students and 58% of pharmacists either agreed or strongly agreed. Granted, this amount would not be expected to actually apply for a combined program, but this does indicate some level of interest on behalf of both future and present pharmacists. From a stakeholder perspective, it was felt that the College of Pharmacy and Nutrition should entertain looking into a combined degree. The responses from these 3 groups are important for program planners and developers, as it is the only known published assessment of demand that we are aware of.

It is important to examine the number of applicants when considering how many students a combined degree program may be able to accommodate. The combined program at the University of Alberta has had 4 students go through the program since 2012, and although the University of Toronto had 5 applicants in its inaugural year, it has not had any students complete the combined program to date (Z. Austin, Leslie Dan Faculty of Pharmacy, University of Toronto, personal communication, 5 March 2015; R. Beaumont, Faculty of Pharmacy and Pharmaceutical Sciences, University of Alberta, personal communication, 11 August 2015). A 2014 graduation survey of pharmacy students in the USA showed that 2% (191/9524) of students graduated with a combined PharmD–MBA degree [12]. At the University of Saskatchewan, the College of Medicine had one student enrolled in the joint program in 2014–2015, while the Western College of Veterinary Medicine had 3 students (C. Schroeder, Edwards School of Business, University of Saskatchewan, personal communication, 1 March 2015). Based on the number of applicants seen with other programs along with our survey results, it is reasonable to assume that there will be interest from a small number of pharmacy students per year at the University of Saskatchewan as well.

Overwhelmingly, there was very little knowledge of combined pharmacy–MBA programs with current pharmacy students, pharmacists, and key stakeholders alike. Further, current pharmacists were generally not familiar with the MBA program at the University of Saskatchewan. This is significant as the College of Pharmacy and Nutrition accepts a significant proportion of its students from both in-province and out of province. Hence, creating awareness and marketing a combined program will become a key factor for any school looking to attract potential applicants. Another important consideration is the financial cost of a combined program for students, and hence it was identified that it is important to have scholarships in place for prospective students so that the tuition amount is not a significant burden when attracting applicants (Z. Austin, Leslie Dan Faculty of Pharmacy, University of Toronto, personal communication, 5 March 2015).

Currently available pharmacy–MBA combined programs are diverse in nature, and provide business-development opportunities for a limited number of students per year. At the University of Alberta, students take time off between 3rd and 4th year pharmacy to enroll in the MBA [13], and University of Toronto students do some courses throughout the pharmacy program and then finish their MBA degree after graduating from pharmacy school [14]. Some schools in the United States of America allow students to attain both degrees at the same time, and recently Washington State University has established a joint program where the MBA program can be taken online; this aligns with their mission to prepare leaders in the pharmacy profession [15]. Hence, there are many ways to deliver a combined program.

With partnerships already established with the College of Medicine and Western College of Veterinary Medicine, the Edwards School of Business is presented an opportunity to offer classes that may be focused on health care administration, particularly as there is still significant interest from current pharmacists in the traditional MBA program as evidenced by our survey results and the number of pharmacists currently enrolled in the MBA program at the University of Saskatchewan. Some external stakeholders felt it was important for a combined PharmD–MBA to have healthcare-related content within the MBA program, and hence this could be seen as an opportunity for the University of Saskatchewan to differentiate itself from the other Canadian schools that either currently offer a combined program or are in the contemplation stage.

Finally, around 50% of pharmacy students indicated they are either interested in owning a pharmacy upon graduation or interested in a career in management. These are the positions many stakeholders felt would most benefit from an MBA degree, and this aligns with the skills MBA programs are trying to develop. Just over half of all pharmacists who responded to our questionnaire felt that an MBA degree would be valuable to their current job, which is significant given that over half of all respondents identified as staff pharmacists not in managerial positions. Combined with the fact that nearly 90% of pharmacists indicated that those students graduating with a combined degree would be at an advantage for career opportunities, this indicates some degree of value which pharmacists place on an MBA degree and how it aligns with pharmacy careers.

## 5. Limitations

The questionnaires distributed were to Saskatchewan students and pharmacists only, hence it is unknown how applicable these results are outside of this province. Also, just less than 20% of all Saskatchewan pharmacists completed the online questionnaire, and so the results are not totally representative of all Saskatchewan pharmacists. Further, although pharmacy students showed significant interest in a combined program, the questions were theoretically based, and it is unknown if interest in a program would translate into actual applications.

The interviews conducted were only with a select number of key stakeholders identified by the researchers, and hence not representative of all stakeholder groups. This could also lead to selection bias. Although there was unanimous support from a high-level overview, it would be appropriate to re-engage these groups as well as a broader scope of stakeholders once program specifics were developed.

## 6. Conclusions

Overall, pharmacist, pharmacy student, and stakeholder input indicate that a combined program could be supported, and the program could likely accommodate a small number of students per year. In order to extrapolate these findings to other pharmacy schools across Canada, each of the other schools should perform their own analysis to determine if pursuing a combined degree is something they want to consider.

## Figures and Tables

**Table 1:** Pharmacist Demographics.

Practice Setting	Number, *n* = 304 (Percent)
Staff pharmacist (community)	110 (36.2%)
Staff pharmacist (hospital)	54 (17.8%)
Pharmacy manager (community)	51 (16.8%)
Pharmacy management (hospital)	15 (4.9%)
Pharmacy owner	44 (14.5%)
Primary care pharmacist	4 (1.3%)
Pharmaceutical industry	3 (1.0%)
Academia	6 (2.0%)
Other (*i.e.*, government, academic detailing, consultant, *etc.*)	17 (5.6%)
**Years since Graduation**	**Number, *n* = 303 (Percent)**
within the past 3 years	38 (12.5%)
3 to 6 years ago	37 (12.2%)
7 to 10 years ago	45 (14.9%)
greater than 10 years ago	183 (60.4%)

**Table 2:** Retrospective interest in a combined pharmacy–MBA degree.

Had it been available in my years as a pharmacy student, I would have been interested in pursuing a combined Pharmacy–MBA degree:	Number, *n* = 301 (Percent)
Strongly Disagree	38 (12.6%)
Disagree	91 (30.2%)
Agree	119 (39.5%)
Strongly Agree	53 (17.6%)
